# Contestable AI for criminal intelligence analysis: improving decision-making through semantic modeling and human oversight

**DOI:** 10.3389/frai.2025.1602998

**Published:** 2025-07-01

**Authors:** Falk Maoro, Michaela Geierhos

**Affiliations:** Research Institute CODE, University of the Bundeswehr Munich, Neubiberg, Germany

**Keywords:** contestability requirements, AI-driven information extraction, named entity recognition, semantic triples, sequence classification, relationship extraction

## Abstract

Criminal investigation analysis involves processing large amounts of data, making manual analysis impractical. Artificial intelligence (AI)-driven information extraction systems can assist investigators in handling this data, leading to significant improvements in effectiveness and efficiency. However, the use of AI in criminal investigations also poses significant risks to individuals, requiring the integration of contestability into systems and processes. To meet this challenge, contestability requirements must be tailored to specific contexts. In this work, we analyzed and adapted existing requirements for criminal investigation analysis, focusing on the retrospective analysis of police reports. For this purpose, we introduced a novel information extraction pipeline based on three language modeling tasks, which we refer to as semantic modeling. Building on this concept, we evaluated contestability requirements and integrated them into our system. As a proof of concept, we developed an AI-driven information extraction system that incorporates contestability features and provides multiple functionalities for data analysis. Our findings highlight three key perspectives essential for contestability in AI-driven investigations: information provision, interactive controls, and quality assurance. This work contributes to the development of more transparent, accountable, and adaptable AI systems for law enforcement applications.

## 1 Introduction

Modern policing faces many challenges, including the increasing complexity and volume of data related to potential criminal activity. Investigators must process vast amounts of information under time constraints, often with limited resources. In the process of collecting, evaluating and interpreting information about such activities for criminal intelligence analysis, the volume of data generated cannot be processed manually (Dubravova et al., 2024). Manual approaches to analyzing such data are often time-consuming and error-prone, limiting the ability to respond effectively to ongoing or emerging threats. In addition, bias, incomplete information, and the potential for human error can hinder the effectiveness of traditional investigative methods. The increasing volume and complexity of data in criminal investigations underscores the need for automated systems to help investigators process and analyze information more efficiently (Du et al., 2020). Such systems can enhance the capabilities of investigative teams by extracting critical information, identifying patterns, and uncovering actionable insights, ultimately improving the accuracy and efficiency of investigations. To ensure reliable and flexible use in sensitive areas, these systems must not only provide robust technical solutions, but also enable users to interact with, track, and correct the decisions they generate. Moreover, such systems and the decisions they make can have a significant impact on the lives of individuals. They should therefore be able to challenge such decisions in order to protect their rights, freedoms and legitimate interests. However, Almada (2019) emphasizes that challenging decisions after they have been made does not always provide strong protection for individuals.

A better approach would be to design systems with *contestability* in mind at all stages of the system lifecycle, allowing for human oversight both before and after key decisions are made. Contestability in this context refers to the ability of stakeholders (e.g., investigators, forensic analysts, legal professionals, policymakers, and potentially affected individuals) to understand, question, and challenge the decisions and outcomes of AI systems. This ensures that the system remains transparent, accountable, and compliant with ethical and legal standards. Henin and Le Métayer (2021) state that embedding contestable systems in decision-making processes leads to more effective outcomes because, among other things, contestability allows users to detect and correct incorrect decisions made by a system. It also makes the use of the system more responsible because a decision can be accompanied by a justification. Moreover, because users are provided with information about a decision and the ability to challenge it, decision makers remain autonomous within this process, as opposed to a system without this ability. Designing for contestability ensures that the outputs of the system are trustworthy and can be critically evaluated. This is particularly important in the context of criminal investigations, where the consequences of erroneous or biased outputs can have serious implications for justice and public confidence. Implementing contestability involves a variety of practices, such as risk mitigation through ex-ante safeguards and third-party (human) oversight, including quality assurance throughout all phases of system development, such as business and use case development, system design, construction, testing, deployment, and monitoring (Alfrink et al., 2023). The complexity of the intertwined requirements for such systems motivates this research. It proposes a process for implementing a prototypical contestable AI system for text analysis.

This paper provides examples of technologies designed to enable contestability by addressing multiple perspectives on how to design systems. We will apply the technical and ethical requirements to the implementation to identify, demonstrate, and resolve the issues as we present a real-world use case in criminal intelligence analysis of police reports. In contrast to digital crimes such as online fraud, this work focuses on analog crimes, including violent, drug, and gun crimes, which often involve physical events and facilities that require thorough investigation. The goal of analyzing this data is to extract knowledge about the events involved. For example, an event might include details about violence, locations, people involved, and associated objects such as weapons or vehicles. Extracting this information from text results in the creation of knowledge that is useful for investigative purposes. A benefit of this analysis is decision support by presenting extracted and analyzed information to investigators. The structured data can provide clarity and focus, enabling law enforcement to more effectively allocate resources and prioritize investigative leads. Proactive investigation involves analyzing data before an event occurs. By identifying criminal activity in its planning stages, authorities can take preemptive action to prevent crime or mitigate its impact. In addition, retrospective analysis of events allows investigators to reconstruct sequences of events, identify persons and entities of interest, and uncover relationships between them. This structured knowledge can be integrated with other investigative tools to help identify evidence and solve crimes.

Our main contributions include a novel approach to extract structured information from police reports by integrating several language modeling tasks, such as multi-label classification, token classification, and relationship extraction, with rule-based methods and external knowledge sources. To facilitate this, we introduce a dedicated annotation scheme tailored to the complexity of police narratives. Furthermore, we develop a prototypical web application that focuses on the principles of contestability, ensuring that users can scrutinize and challenge the system's results. Beyond the technical aspects, we analyze the requirements, stakeholders, and challenges associated with contestability in police software and propose concrete solutions to enhance transparency and accountability.

To do so, we first review the existing literature in Section 2.1, which covers existing contestability requirements, AI tools in police work, and information extraction with large language models. The problem and research gaps are then described in Section 2.2, before the system design approach is presented in Section 2.3. This section describes the data collection, processing, and annotation techniques used to train and evaluate the AI system. It also presents the conceptual design of the system, which integrates semantic modeling with human supervision to support effective decision-making. In Section 3.1, we adapt the contestability requirements by applying them to our use case of criminal intelligence analysis and the stakeholders involved in our prototypical system. The adapted contestability requirements are the basis for Section 3.2, which evaluates the models, and Section 3.3, which presents the prototype integration and the contestable system features. Finally, Section 4 discusses the results in the context of the limitations of the approach and presents further research.

## 2 Materials and methods

Designing an information extraction system that works with potentially sensitive data in the context of criminal intelligence analysis can raise ethical concerns about proper behavior. One approach to mitigating such concerns is to design with contestability in mind. We do this by first reviewing the literature in Section 2.1, then specifying the problem statement in Section 2.2, and finally proposing a concept for an information extraction system for police-relevant data in Section 2.3.

### 2.1 Related work

The development of a contestable AI system that supports police investigators in extracting structured knowledge about events from textual datasets requires the consideration of several aspects. Since contestability is the main criterion for the quality of this system, Section 2.1.1 focuses on available resources regarding contestability requirements, the development of contestable systems, and frameworks. In addition, AI tools in policing, including their successes and limitations, are covered in Section 2.1.2. Finally, Section 2.1.3 highlights the use of large language models to extract structured information from unstructured data.

#### 2.1.1 Contestability requirements

Contestability in AI systems, as introduced earlier, can be approached from different perspectives, implying requirements for all stages of the development life cycle, algorithms, user interface components, and the structure of decision processes. In the following, we examine the most prominent perspectives, comparing and, where possible, merging the requirements proposed in the perspectives.

Alfrink et al. (2023) presents a framework for developing contestable AI systems that combines different perspectives on contestability. It presents practices that enable contestability in the AI lifecycle and maps system characteristics to stakeholders in the AI lifecycle. Stakeholders include system developers, human controllers, decision subjects, third parties, and the AI system itself. The characteristics and practices provide a taxonomy of contestability requirements. In the following, we use this taxonomy to examine existing requirements and approaches to meeting these requirements in more detail.

General features that enable contestability include *built-in safeguards against harmful behvaior, interactive control over automated decisions, explanations of system behavior, human review and intervention requests*, and *tools for scrutiny by subjects or third parties*.

The first feature, *built-in safeguards*, is created by system developers and constrains an AI system by limiting the effects that an AI system's decisions can have on decision-making processes. This could be a check mechanism using another system or human reviewers (Alfrink et al., 2023). In addition, all decisions and changes can be recorded, allowing for monitoring (Almada, 2019).

*Interactive controls* are used by decision subjects and human controllers (Alfrink et al., 2023). They allow a system's decisions to be corrected or overridden (Hirsch et al., 2017; Bayamlhoğlu, 2022).

Hirsch et al. (2017) requires that a system be designed to have comprehensible AI models and results that users can understand. This can be done by highlighting and explaining measures such as confidence scores that allow users to track predictions and challenge the reasoning if necessary. Moreover, key indicators of a decision, alternatives to a decision, and information about counterfactuals are needed to allow users to make informed decisions about the validity of a decision (Ploug and Holm, 2020). *Explanations of system behavior* provide decision subjects and human controllers with information to verify a system's reasoning and to understand automated decisions (Lyons et al., 2021; Alfrink et al., 2023).

The next feature contains requirements regarding *human review and intervention requests*, which primarily focus on decision subjects and third parties to challenge decisions and record interventions (Alfrink et al., 2023). Ploug and Holm (2020) emphasizes the right to contest the use of personal data. Since user data may be processed, a user must be informed of what data is being processed and the sources of that data in order to decide whether to consent to that processing. Another approach to human review is proposed by Almada (2019), who suggests that the system should present multiple choices and require the human to actively select an option for decisions. This approach ensures that human decision makers apply their prior knowledge and values, thus promoting a more meaningful and responsible decision-making process. Henin and Le Métayer (2021) introduce a challenge-justification framework and apply their approach to a credit decision system, demonstrating how users can challenge decisions based on norms and justifications. Statements, which are preliminary decisions, can be challenged by referring to norms, rules, or principles. Rationales are then used to defend or revise a decision based on evidence. This allows for an iterative process of challenge and justification until consensus is reached. Finally, to intervene in biased decisions or abuse, systems should allow users to provide feedback, register disagreements, and flag potential problems, which should be used for continuous improvement leading to fairer outcomes (Hirsch et al., 2017).

*Tools for scrutiny by subjects or third parties* of AI systems provide information resources to external actors such as decision subjects, indirect stakeholders, and third parties (Alfrink et al., 2023). These may be non-functional requirements, such as detailed, accessible documentation for users to obtain all relevant information of a system (Almada, 2019). Ploug and Holm (2020) presents four dimensions of information provision. The first dimension concerns the type and source of data. The second dimension concerns information about bias against sensitive attributes such as gender, age, or ethnicity. Information must be provided about the nature of training and evaluation data, human annotations, and the nature of model testing with respect to bias. Although system errors are inevitable, information about the performance of a system, the third dimension, such as measurements, lends credibility to the system and makes the individual decision contestable. Finally, the fourth dimension is information about the embedding of AI systems in decision-making processes. Although AI-based systems can improve the quality of decision making, when embedded to support humans, this can lead to over-reliance on such systems. Therefore, stakeholders should be informed about how AI is embedded in a decision-making process and how objective and legal responsibilities for such decisions are defined.

The categories of practices that lead to contestability include *ex-ante safeguards, agonistic approaches to machine learning development, quality assurance during development and after deployment, risk mitigation*, and *third-party oversight* (Alfrink et al., 2023).

*Ex-ante safeguards* involve stakeholders in early life cycle phases to anticipate the impacts of a system in advance (Alfrink et al., 2023; Henin and Le Métayer, 2021). By adding contestability as a requirement, contestability must be evaluated for the context of a system by defining what is contested, who can contest, who is responsible, and how contestability is implemented (Lyons et al., 2021; Alfrink et al., 2023). This also requires involving stakeholders in the process to identify potential threats to rights and interests (Almada, 2019).

Another early-stage practice considers *agonistic approaches to machine learning development*, leading to decision systems that do not rely solely on AI algorithms (Alfrink et al., 2023). Kariotis and Mir (2020) emphasize participatory design that invites stakeholders to influence key design decisions that can be integrated through ongoing dialogue. Decision processes and the role of humans in automated decision making should be carefully designed. If humans are only tasked with overseeing automated decisions by confirming or correcting them, there is a risk of poor decision making and reduced autonomy, as this limits their engagement in critical evaluation and independent judgment (Almada, 2019).

*Quality assurance during development* involves measuring performance against multiple objectives. This can be bias measurements or accuracy measurements (Hirsch et al., 2017; Ploug and Holm, 2020; Alfrink et al., 2023). Hirsch et al. (2017) argue that poor system performance discourages user adoption, increases the workload of contestation by requiring constant corrections, and undermines trust in the system, which ultimately reduces credibility and usefulness.

After deployment, the *quality assurance* must continue. Biased data or human inputs through intervention can lead to algorithmic discrimination against sensitive attributes within a system. Detection of bias should therefore be done by monitoring automated and human-corrected decisions in combination with sensitive attributes (Almada, 2019). Thus, monitoring decisions in combination with such attributes can lead to the detection of bias.

*Risk mitigation strategies* concern risks about a system context and the users (Alfrink et al., 2023). Hirsch et al. (2017) suggests that the design of *training programs* for users should explain the capabilities, strengths, and weaknesses of the system and equip them to critically evaluate and challenge its decisions.

The final practice presented by Alfrink et al. (2023) is *third-party oversight*, which should be omnipresent throughout the lifecycle of the system. It ensures that that systems comply with rules and regulations (Bayamlhoğlu, 2022).

#### 2.1.2 AI in policing

Policing is multifaceted, encompassing investigation, intelligence, prevention and mitigation, public protection, and security assessment. Within these areas, information systems help authorities gain insight into data from multiple sources, resulting in efficient and effective processes. Information systems store data, provide analytical tools, can recommend actions, assess risks, and allow users to access accurate information. Due to the significant amount of data, more and more systems are using AI algorithms. Examples of the use of AI include predictive policing, where crime patterns are analyzed to make spatial or personal predictions about the occurrence of future crimes (Povalej and Volkmann, 2021). Such systems work with data that contains information about people, their behaviors and characteristics. There is a risk that a system will make unwarranted decisions based on inappropriate characteristics, such as sensitive attributes like race or gender. In this context, assessing the fairness of such systems is imperative. For example, there is a variety of work examining the fairness of algorithms for predictive policing algorithms (Alikhademi et al., 2022; Ziosi and Pruss, 2024). Angwin et al. (2016) found evidence of a system disadvantaging people of color in a prediction system for assessing a defendant's risk to commit a future crime. Other AI-enabled policing processes include facial recognition (Guo and Kennedy, 2023; Simmler and Canova, 2025), deepfake and misinformation detection (Wang et al., 2024; Liu et al., 2024), or crime analysis of social media, online content, or police reports (Hajela et al., 2020; Ates et al., 2021).

For retrospective criminal intelligence analysis, there is research that examines police reports. Carnaz et al. (2020) developed a pipeline for sentence-wise named entity recognition on Portuguese police reports. They used four types of entities: persons, places, organizations, and dates. In addition, Carnaz et al. (2019) present a framework for extracting relationships from Portuguese police reports. The proposed framework works with part-of-speech tags, lemmatized words, and named entities to populate an ontology that represents the relevant knowledge extracted from the criminal police reports.

Since existing work on information extraction systems in the context of criminal intelligence analysis does not focus on contestability, this work attempts to fill this gap. Although work exists that considers retrospective analysis of police-relevant data, we build on this work by expanding the variety of extracted information, improving the effectiveness by using state-of-the-art methods, and using the concept as a basis for designing and implementing a contestable AI system.

#### 2.1.3 Large language models for information extraction

Information extraction enables the automatic transformation of unstructured textual information into structured information by using natural language processing (NLP) techniques (Pazienza, 1997). Textual data can contain a variety of useful information in different contexts. NLP techniques implement algorithms that process textual data to solve tasks involving the identification and classification of relevant information. For example, sequence classification assigns labels to an input sequence in a binary, multi-class, or multi-label setting using feature- and rule-based classifiers or statistical models (Xing et al., 2010). Other techniques include the analysis of linguistic features through part-of-speech tagging (Ratnaparkhi, 1996), topic modeling with vector representations of text sequences (Mikolov et al., 2013), or named entity recognition (NER) (Nadeau and Sekine, 2007). NER is a form of token classification that assigns classes corresponding to real-world meanings to spans, which are sequences of text consisting of one or more tokens. For example, the entity class *LOCATION* indicates that a text span contains information about a location. This task can be adapted to arbitrary labeling schemes, allowing the extraction of text strings and their corresponding labels, e.g., for persons, organizations, time information or bank details. Building on the classified tokens and entities, the relationship extraction task can identify relationships between pairs of identified entities within a text sequence (Han et al., 2020). Relationships can be used to model real-world concepts and can therefore indicate relationships such as who was where, who is a parent and who is a child. By combining the NER and relationship extraction tasks, complex contexts and domains can be modeled to provide structured information about textual content.

Traditionally, NLP applications have relied on rule-based systems and statistical machine learning models. Significant performance improvements have been achieved through the introduction of deep learning algorithms and the Transformer architecture, which is the basis for several state-of-the-art large language models (LLMs) (Vaswani et al., 2017). LLMs such as BERT, RoBERTa, or T5 leverage pre-training on large datasets and can be reused for fine-tuning on multiple information extraction tasks, while requiring comparatively small amounts of labeled data and demonstrating superior generalization capabilities (Devlin et al., 2019; Liu et al., 2019; Raffel et al., 2020). These models operate on text that has been tokenized and converted into numerical representations. Tokenization splits text into subword units or tokens, each mapped to a unique identifier. The resulting sequence of token IDs captures information about word content, order, punctuation, and other linguistic features, enabling the model to learn complex patterns and nuances of language. One limitation of these models is their fixed context length, which restricts the number of tokens that can be processed at once. As a result, very long text sequences may be truncated or require special handling to avoid loss of information.

Selecting a model for a task should be done by evaluating several model characteristics, such as performance on benchmark datasets, model size and hardware requirements, pre-training data, and language capabilities, relative to task complexity, available resources, and performance goals.

### 2.2 Problem

Research on contestable AI systems for criminal intelligence analysis is still in its early stages. While previous work has independently explored information extraction techniques, contestability requirements and frameworks, and the use of advanced language models for NLP tasks, several challenges remain, especially when combining these approaches. One limitation of existing work is the narrow scope of the information extraction implemented. Existing approaches focus on a limited range of data types, thereby restricting the depth and utility of automated analysis. In addition, many police systems offer only basic analytical capabilities and lack the flexibility needed for complex investigations and case studies. Another critical issue is the lack of contestability mechanisms. While general frameworks exist, their application to criminal intelligence analysis remains unexplored, making it difficult for users to question or correct AI-driven decisions. Furthermore, existing systems rely on outdated information extraction techniques, resulting in suboptimal performance compared to recent advances in LLMs. Overcoming these challenges is essential to developing more effective, transparent, and adaptable systems for analyzing police reports.

Since there is no well-defined implementation for contestability in criminal intelligence analysis, our research aims to fill this gap by exploring how contestability requirements should be designed, adapted, and implemented, taking into account both system capabilities and user needs. Therefore, we pose the first research question:

**RQ1**: For an AI-based criminal intelligence analysis system, how must contestability requirements be adapted?

To answer this question, we plan to design and implement a criminal intelligence analysis system. The specific system builds on existing research on police report analysis. Therefore, we want to expand the scope of information extraction in this analysis by extending the range of data types that can be identified and analyzed. This goal leads to a second research question:

**RQ2**: How can police reports be modeled to support investigative work?

The system should provide both an insightful analysis tool and contestability solutions to the adapted requirements. Therefore, in the following we present our concept of semantically modeling police reports, before adapting contestability requirements and implementing a contestable AI system.

### 2.3 Concept

Since we want to adapt contestability to AI-based criminal intelligence analysis, we present a concept for an information extraction pipeline and a system that uses this pipeline to provide an interactive user interface. This concept serves as a basis for the subsequent adaptation of contestability requirements to the concept, the implementation of a proof of concept, and the evaluation of the quality of the information extraction.

To develop this automated criminal intelligence analysis system, several interacting components are required. Since the system is based on data to be analyzed, this data must be processed and stored before it can be made available for exploration in a user interface. One approach to develop such a system is to design a distributed system that includes encapsulated services (Verissimo and Rodrigues, 2012). Our proposed system includes two main services: a *data processing pipeline* and a *user interface*.

**Figure 1:**
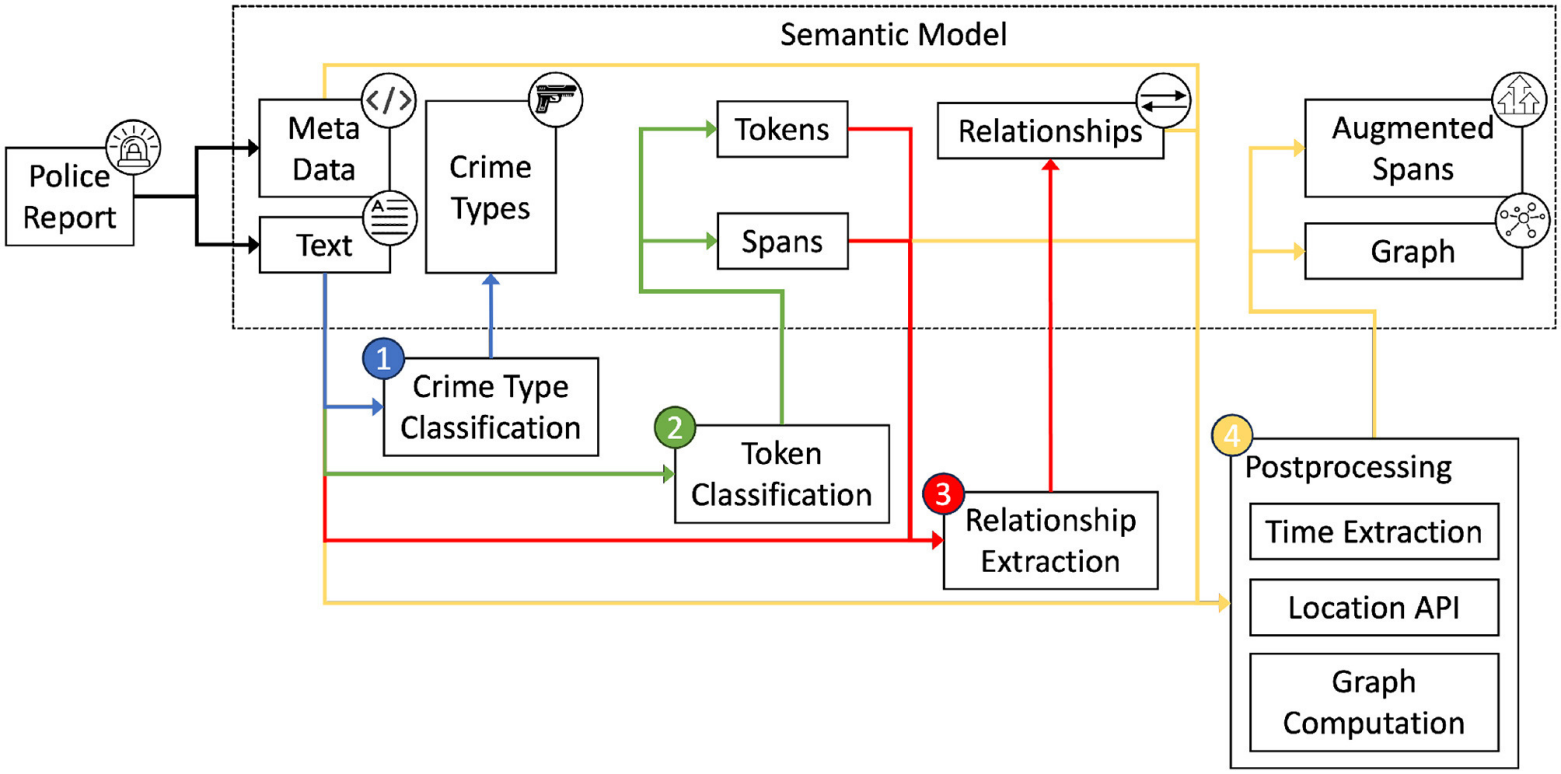
Overview of the semantic modeling process.

The *data processing pipeline* receives available data, extracts relevant information, and builds a semantic model. To extract structured information from the data, the pipeline combines the results of three language modeling tasks and a post-processing step to form this semantic model. An overview of the semantic modeling process is shown in Figure 1. The police report contains the content and metadata that are the basis for all further operations. The pipeline consists of four steps. The first step (1), crime type classification, predicts the crime types present in the text and stores them in the semantic model. The next step (2), token classification, tokenizes the text, classifies each token, and creates entity spans. The third step (3), relationship extraction, uses these spans to classify the relationships between them. After the three language modeling tasks, a post-processing step (4) is performed. Here, the extracted spans are enriched with structured information from time extraction and location query libraries. In addition, a graph is computed using a specialized parsing algorithm.

The *user interface* allows users to interact with the data and the pipeline and implements the contestability requirements. Most important for this user interface is the ability for users to interact with intermediate pipeline steps to challenge decisions and thus modify pipeline outputs.

This section presents the pipeline concepts and a prototypical application. The basis for the pipeline that creates the semantic model is a dataset, which is introduced in Section 2.3.1. Using this data, three language modeling tasks are presented. The first task, sequence classification, is presented in Section 2.3.2. Here, the types of crimes contained in the described events of a data instance are classified. In Section 2.3.3, another language modeling task, token classification is introduced. This allows the extraction of entities such as persons, locations, or objects. Some of the extracted entities have relationships to each other. The classification of these relationships is done in Section 2.3.4. The final component of the pipeline, presented in Section 2.3.5, combines the previous three steps. By aggregating the input data, metadata, and extracted information from the three tasks into a semantic model, a unified data model is created for further use in the police investigation process. Finally, our approach for implementing a prototype system is presented in Section 2.3.6.

#### 2.3.1 Data

**Figure 2:**
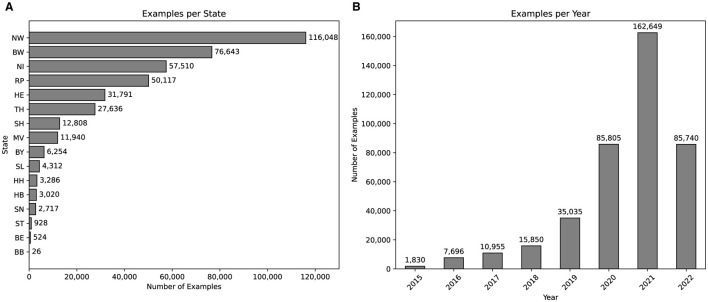
Visualization of dataset statistics. **(A)** Number of examples per state. **(B)** Number of examples per year.

Police authorities often publish reports on recent operations and events. These reports provide transparency for police work and allow news agencies to disseminate information to a wider audience. One platform where German police authorities publish is *https://www.presseportal.de/*. The platform offers a category for police reports, which contains police reports from several German police authorities spread across the country. Each entry contains a number of data points, including a headline, time and date of publication, publisher, tagged locations and topics, textual contact information for the publisher, and the written police report. A custom web crawler was used to generate a raw dataset of 405,560 examples. There are 14,632 different topics and 38,523 different locations tagged. Since Germany has 16 federal states (BB, BE, BW, BY, HB, HE, HH, MV, NI, NW, RP, SH, SL, SN, ST, TH), we have extracted the states from the tagged locations. The distribution, as shown in Figure 2A, is characterized by unevenness, with certain states, such as *NW* or *BW*, showing a significant overrepresentation compared to others, including *BB* or *BE*. While this could be interpreted as if there was a large imbalance in terms of criminal activity between different states, it merely shows the amount of reports published in those states on the given platform. Moreover, the distribution of examples per year in Figure 2B shows a similar unevenness. This distribution indicates a significant increase in published police reports after the year 2016. It should be noted that there are no examples for the second half of the year 2022, which explains that the number of examples decreased by half compared to the year 2021.

As mentioned earlier, the data serves as the basis for the semantic modeling pipeline, which is shown in Figure 1. In the following, three language modeling tasks are presented. In order to train language models on these tasks, the presented data will be further analyzed, preprocessed and annotated.

#### 2.3.2 Sequence classification

The first task in the semantic modeling pipeline, shown in Figure 1, takes the text of the police report, tokenizes it without further preprocessing, classifies it with a language model, and stores the predicted labels in the semantic model. Each report and each reported event contains one or more crimes. Identifying the crime types in a report at a glance, without having to read the entire report, saves time and allows filtering large datasets to relevant subsets. Manual inspection of police reports led to the decision to classify four broad crime types. The first crime type, *drugs*, refers to all crimes related to drug trafficking, possession, or production. The second type of crimes, *weapons*, is related to events, which deal with any form of unauthorized possession, threatening or use of weapons. The third type of crime is called *danger to life and limb*. This includes all reports that involve some form of violence against others. The final type of crime, *other*, covers all crimes that do not fall into any of the previous three categories, such as theft, fraud, or property damage. Although the level of detail and validity of the selection of these four categories can be debated, they were chosen for their unambiguousness and separability, which allows for precise and quick annotation in the labeling process. In addition, the reports mostly deal with clear police actions and closed incidents, rather than ongoing investigations into, for example, intangible incidents, online fraud, or insults. Therefore, the number of crime types available in this dataset is limited compared to all possible real-world crime types.

For this sequence classification task, we only use police report texts as sequences without any modifications. Fine-tuning a language model on this supervised task with the given labels requires annotated data. Therefore, we annotated the crime types with three annotators and split the data into a training and a validation split using a 70/30 ratio. The number of labels per split is shown in Table 1. The dataset contains 758 examples with an unbalanced distribution of labels. The proportion of true labels in the whole dataset ranges from 18 % for weapons to 54 % for drugs, which could lead to uneven performance of a classification model for different classes.

**Table 1:** Label distribution of crime types across training and validation splits, as well as the entire dataset.

	**Train**		**Validation**		**Sum**	
**Category**	**True**	**False**	**True**	**False**	**True**	**False**
Drugs	299	232	115	112	414	344
Weapons	106	425	36	191	142	616
Other	188	343	101	126	289	469
Danger to life and limb	164	367	74	153	238	520

#### 2.3.3 Token classification

Classified crime types mentioned in a police report convey limited information about the events mentioned. To extract more details, the second step, as shown in Figure 1, classifies text segments, called tokens, assigns them labels that correspond to the type of information, and combines them to spans. To do so, the text sequences are, again, tokenized without any modifications, before being passed to the language model.

Police reports contain unstructured information about people, groups of people, objects, locations, times and dates, and relationships created by associations or actions within the events described. Understanding this unstructured information as an investigator is done by reading and possibly taking notes, which is time-consuming because it requires an investigator to focus on one text at a time. In addition, the information within the reports, or only a subset of the information, is needed for further investigation or comprehensive analysis processes within authorities. One approach to extracting this information is to classify tokens into predefined information categories. By using predefined categories, relevant data can be extracted and stored in databases, enabling filtering for specific values or automated analysis processes.

There are few labeled German data and thus few German models with a large number of different token classes. Therefore, our approach is to define an extended classification scheme, manually label our data, and train a token classification model. The most common classification scheme for this task contains four different span classes: *LOC, MISC, ORG*, and *PER* (Tjong Kim Sang and De Meulder, 2003). As a starting point, we used a German NER model, *flair/ner-german-large* (Schweter and Akbik, 2021), trained to predict these four span classes, to predict our data. The analysis showed that hardly any *PER* spans were present in the reports. Since there are other relevant information types within the reports, we defined a labeling scheme consisting of 7 different classes, modifying this existing scheme. The first class, *LOCATION*, is taken from previous research and refers to any form of location, such as countries, cities, streets, or specific named places. The next label, *ORGANIZATION*, is also adopted and refers to companies, institutions, government bodies, and other formal organizations. Since there are no people names in the police reports, people and groups of people are referred to by terms such as “the perpetrator”, “the fugitive”, “he”, “she”, “they”, or “the employee”. For these referring terms, we define the label *PERSON-REFERENCE*. When a group of people is mentioned in text, the group is usually preceded by a counting term or number, such as “the three bikers”. We define the label *COUNT*, which refers to these counting terms. Counting terms are also used to specify amounts and units of objects. In this context, objects can be any item relevant to the event. Examples are terms for weapons, drugs, vehicles, or clothes. We define the label *OBJECT* for these terms. Police reports are descriptive and as such use descriptive terms to characterize people and objects. The label *PROPERTY* refers to such descriptive terms as colors, descriptions of shape, size, or age. The last label, *TIME*, refers to all time or date specifications.

Since the aforementioned German NER model is capable of predicting two of our defined labels, namely *ORGANIZATION* and *LOCATION*, we used the model to predict these labels before manually correcting the predicted labels and adding the labels from our custom scheme. We also used the *duckling* library (available at *github.com/facebook/duckling*) to predict *TIME* labels. The library implements regular expressions for a number of dimensions, such as time, email, quantity, or volume, in different languages. For *TIME* annotations, this also provides standardized time and date values, which helps in automated processing of the data. The manual annotation and correction process resulted in 751 examples. The distribution of labels is shown in Table 2.

**Table 2:** Label distribution in the training and validation datasets.

**Label**	**Train**	**Validation**	**Sum**
COUNT	4,795	607	5,402
LOCATION	3,281	386	3,667
OBJECT	4,748	500	5,248
ORGANIZATION	1,400	156	1,556
PERSON-REFERENCE	8,383	1,060	9,443
PROPERTY	4,126	366	4,492
PERSON	55	8	63
TIME	2,129	203	2,332

The *TIME* and *PERSON* labels were excluded from the training of the token classification models.

#### 2.3.4 Relationship extraction

In Section 2.3.3, we developed a model to extract a custom scheme of spans from the police reports available in our data. Although the extracted information is structured, its value is limited to a standardized format for *TIME* spans, the exact position, and the type of information it refers to. To increase the value of the information extracted from the span labels shown in Table 2, relationships between the identified spans can be extracted. This third step of the semantic modeling pipeline, shown in Figure 1, takes the unmodified text sequence and the corresponding token classification outputs, namely tokens and spans, and extracts relationships by classifying all potential relationship combinations within the police report. This gives context to the spans and allows further analysis. For example, if there are multiple *PERSON-REFERENCE* spans and multiple *LOCATION* spans, it would be important to know which person was in which place.

In the real world, there are an infinite number of possible relationships. However, it is impractical to model the entirety of the nearly infinite number of relationships within the scope of this study. Therefore, we analyzed the existing annotations for spans and the possible relationships between them to define a scheme that fits the use case of extracting information from police reports. The resulting annotation scheme contains 5 possible relationships between different spans of Section 2.3.3. There are both bidirectional and unidirectional relationships between spans. An overview of all possible relationships is provided in Table 3.

**Table 3:** Possible relationships between spans as source spans (left) and target spans (top).

**Source span**	**Target span**						
	**COUNT**	**OBJECT**	**PERSON-REFERENCE**	**ORGANIZATION**	**TIME**	**LOCATION**	**PROPERTY**
COUNT		COUNTS	COUNTS	COUNTS		COUNTS	
OBJECT		COREF CONTAINS	BELONGSTO				
PERSON-REFERENCE			COREF CONTAINS		STAY	STAY	
ORGANIZATION			CONTAINS	COREF CONTAINS	STAY	STAY	
TIME					COREF		
LOCATION						COREF CONTAINS	
PROPERTY		BELONGSTO	BELONGSTO			BELONGSTO	

The first relationship, denoted by *STAY*, is unidirectional and originates from either *PERSON-REFERENCE* or *ORGANIZATION* and targets *LOCATION* or *TIME*. The *STAY* relationship indicates where people or groups have been or at what time and date they have been somewhere.

The second relationship identified is the *COUNTS* relationship, which is characterized by its tail being a *COUNT* span. It targets *OBJECT, PERSON-REFERENCE, ORGANIZATION*, and *LOCATION* spans. This can be relevant to several automated analysis tools. For example, counting the amount of a drug someone is carrying would allow an automated decision to be made about whether a drug possession limit has been violated. In a comprehensive analysis tool, this data could be used to evaluate the volume of items such as specific weapons, drugs, or money that occur within a city, within reports of a specific crime type, or within a specific time period. In addition, *PERSON-REFERENCE* spans sometimes refer to groups of people. It can be critical to know the exact number of individuals within a given group. If a group of suspects is evading law enforcement, the exact number of people is necessary to track the progress of a potential manhunt. The relationship is less common for places and organizations to be counted, as they are usually unique.

*PERSON-REFERENCE* spans are somewhat ambiguous because such references can be both group mentions and single person mentions. Often, individuals are referenced multiple times within a report by both individual mentions and group mentions. Moreover, their incoming and outgoing relationships with other spans are distributed throughout the report, meaning that the first relationships may be linked to the first mention, while the next relationship may be linked to the second or a later mention. Since these references belong to a single entity, the relationships from multiple references must be merged into this single entity. We do this by using *COREF* relationships, which indicate a coreference of a single entity between multiple references. These relationships are undirected and allow for the clustering of references. In addition to *PERSON-REFERENCE* spans, *OBJECT, ORGANIZATION*, and *TIME* spans can have coreferences to spans of the same class.

Another relationship, *CONTAINS*, indicates that one span includes another span. This could be a broad description of a location that contains a more specific span for that location, an organization that contains another organization, a person or group of people, an object (such as a bag) that contains another object, or a group of people that contains a single person.

The last relationship, *BELONGSTO*, is used when an *OBJECT* span belongs to a *PERSON-REFERENCE*, or when a *PROPERTY* describes an *OBJECT*, a *PERSON-REFERENCE*, or a *LOCATION*.

The annotation scheme contains five possible relationships, each with different source and target spans, resulting in a complex task. The concept provides the most relevant information and ignores rare relationships or edge cases, which limits the scope of manual annotation for the proof of concept. Although there are few types of relationships, the large number of possible relationships in police reports can lead to errors, requiring humans to challenge and correct imperfect predictions.

**Figure 3:**
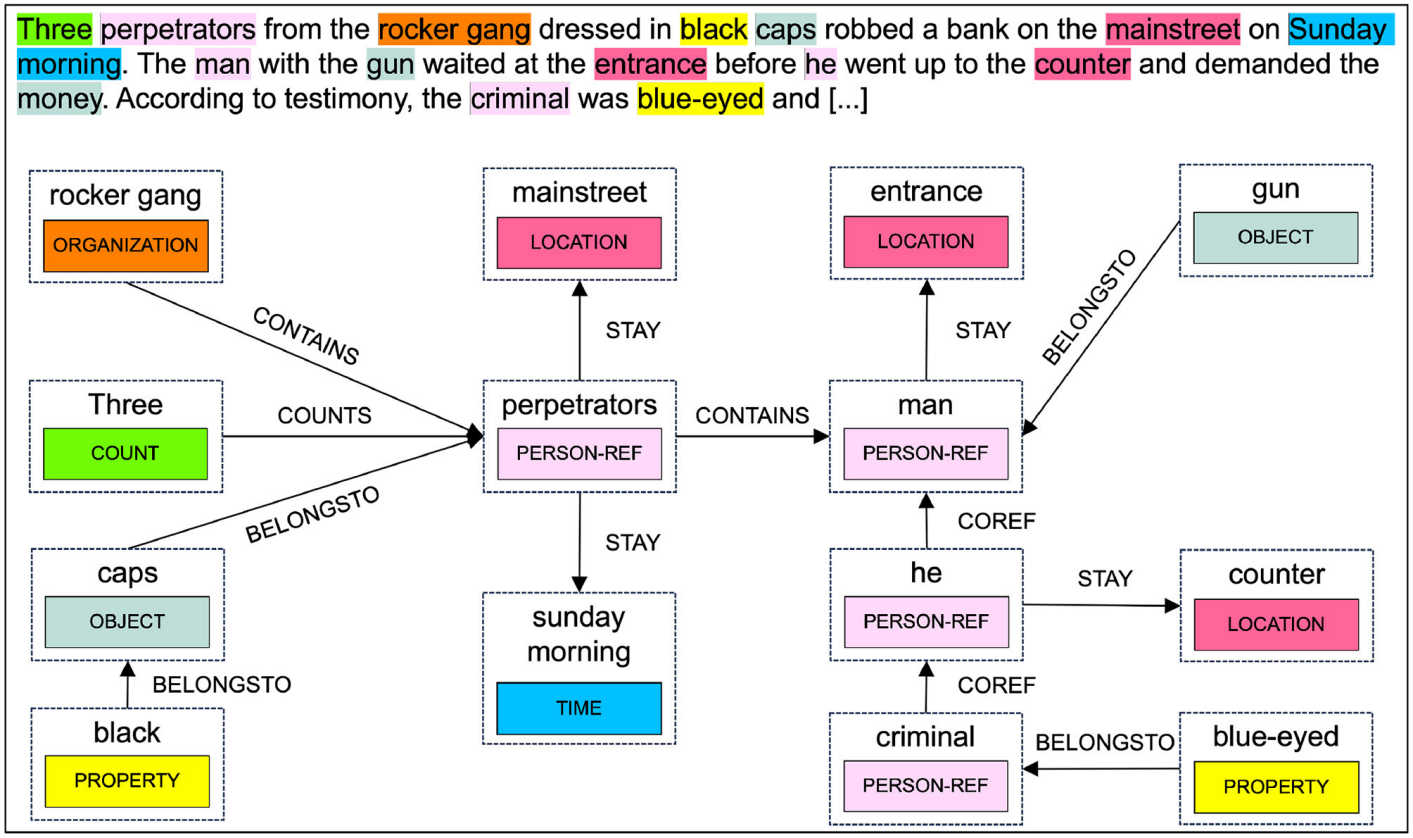
Police report with annotated spans and relationships.

An example showing the available token classes as spans and the corresponding relationships of a police report is illustrated in Figure 3.

Using this labeling scheme, we manually annotated 751 examples, out of which we used 85 % for training and 15 % for validation. Table 4 shows the number of annotated relationships per dataset. Notice that the *BELONGSTO* and *COREF* relationships are about twice as common as the other relationships, resulting in an uneven distribution of labels.

**Table 4:** Number of labeled relationships for the train and validation datasets.

**Relationship**	**Train**	**Validation**	**Sum**
BELONGSTO	7,142	987	8,129
CONTAINS	2,943	541	3,484
COREF	7,146	1,481	8,627
COUNTS	3,781	723	4,504
STAY	3,350	660	4,010

#### 2.3.5 Semantic modeling

The tasks of crime type classification, token classification, and relationship extraction are useful on their own, but they can be combined with external data and metadata to add value to criminal intelligence analysis. This combination of tasks with post-processing is represented by the fourth and final step in the semantic modeling pipeline shown in Figure 1. To store the information resulting from the various pipeline steps, we conceptualize the semantic model, a relational database model that serves as the basis for user exploration and challenge of all intermediate steps. We propose a concept for the semantic model by analyzing all pipeline outputs, the requirements for modification and versioning, and the intention to export information to other formats such as graphs.

Our proposed semantic model is based on police reports, metadata, annotations made by humans or predicted by models, and external data. A semantic model instance contains the textual police report, a dataset ID, and metadata that includes the URL, publication date, tagged locations, and topics, among others. Moreover, a semantic model has three lists of annotation layers. Each list corresponds to the annotations of a task of sequence classification, token classification, and relationship extraction. This abstraction allows to keep multiple versions of annotations per police report. Each version is editable to support contesting AI decisions. There is a special restriction for relationship extraction annotations. Because a relationship requires two spans, deleting or manipulating one span would invalidate the corresponding relationship. To mitigate this effect, relationship extraction annotations are tied to specific versions of token classification annotations. In addition, manipulation of token classification annotations, which includes adding, editing, or removing classified tokens, always creates a new version of that annotation if there is a corresponding relationship extraction annotation. This also implies that removing a token classification annotation will remove the relationship extraction annotation.

Furthermore, token classification annotations are enhanced by aggregating them with metadata and using external data. *LOCATION* spans often contain information about the exact locations where events took place. This can be streets, cities, landmarks, or vague descriptions. Most of the terms used to describe such places can be found in proprietary location data. Geolocation services allow users to enter search queries and find detailed location information for a given location. We use such services to query all *LOCATION* spans. In some cases, for example if a street name exists in multiple cities, multiple hits will be returned for the query. In this case, adding more location information to the query may improve the search result. Because the metadata for police reports sometimes includes tagged locations, such as city or state names, these tagged locations are used to refine the query. The results are then saved with the appropriate span and can be used for further visualization or analysis.

Another way to improve the semantic model is to use the publication timestamp in the metadata. The time expression extraction library can accept a reference time. When parsing a relative time expression, such as “yesterday”, the timestamp is calculated relative to that reference time. This increases the precision of the extracted date and time information, allowing precise analysis of events over time.

The data in the semantic model can be parsed to create a graphical representation of all spans and relationships. This can involve the merging of *COREFERENCE* relationships between multiple *PERSON-REFERENCE* or between multiple *TIME, LOCATION*, or *ORGANIZATION* spans. This process creates clusters of spans that refer to the same entity. Therefore, when accessing the cluster, all relationships from different spans within a cluster can be accessed.

#### 2.3.6 Prototype system

The semantic modeling pipeline shown in Figure 1 includes several processing steps that result in a unified data format, the semantic model. Developing this pipeline requires fine-tuning the language models to the three language modeling tasks, evaluating the quality of the information extraction, and implementing the semantic model, which then allows answering **RQ2** how police reports can be modeled to support investigative work.

Based on this implemented pipeline, we design a proof of concept for a contestable AI system that works with the semantic modeling pipeline. Here, we describe the use case and the stakeholders of this proof of concept, before the contestability requirements are adapted to this system in Section 3.1 and the implementation, integrating the adapted requirements, is done in Section 3.3.

The reports on which the system is based on do not contain personal information of named individuals. The subjects mentioned are not referenced by name or by pseudonyms that would be directly traceable to identified individuals. In addition, the police reports are event-oriented, describing events that occurred, rather than subject-oriented, which would indicate that the reports focus on describing people and subjects of investigation. The information extracted is intended to support the investigative process. In terms of the decision-making process, this means that there is no decision that directly affects a person, as would be the case with a conviction, such as whether or not a person should receive a sentence. The decisions that this system automates concern whether certain parts of the police report should be classified as relevant information and, if so, what type of information that is. Although the extracted information can be used for further investigation and analysis, which could potentially lead to a subject-related decision concerning the rights and freedom of the subject, we argue that this system does not provide such critical decisions. This is also underlined by the fact that there is no automation of the use of the extracted information in the police investigation, as the extracted information must be manually assessed and transferred to the investigation by a responsible user.

Defining contestability requirements for this system requires a characterization of stakeholders. We do this characterization by using the four stakeholder groups presented in Alfrink et al. (2023). *Human controllers* are police investigators who are the primary user group working with the system. They use the system to find relevant information for criminal investigations. They investigate one report at a time and need quick access to the data and functions to correct results, which is best provided by an intuitive user interface. They also need to understand the system's processing structure and the reasoning behind its decisions, because they will be held accountable if they have to provide evidence to support charges based at least in part on the system's results. *Decision subjects*, as argued earlier, are not directly affected by the decisions generated by the system. Therefore, the requirements for decision subjects are rather low. In a legal process, the investigators take responsibility for the allegations and evidence. Decision subjects in this context would not challenge the decisions of the system, but rather the decisions of an investigator. *System developers* are required to provide a system with the highest possible performance and usability. To train language models with sufficient performance, they need high-quality data. This data must be annotated according to precise guidelines and standards, preferably contain uniformly distributed class labels, and ideally be free of bias toward sensitive attributes. Finally, *third parties* include government agencies, law enforcement, or civil society groups. Although system operators, in this case police authorities, may wish to keep data, models, and system internals secret, Bayamlhoğlu (2022) argues that third parties could act as trusted intermediaries for ex ante inspection of the system and *post hoc* challenge of decisions. Thus, information about the system and information about individual decisions should be made available.

Before evaluating the detailed requirements in Section 3.1, minimal contestability requires that users be able to track decisions predicted by language models and make corrections that reactively trigger all relevant processing and visualization steps. To provide these features, we design a modular web application, a distributed system, that assigns the responsibilities for visualization, semantic modeling pipeline processing, language model deployment, and data persistence to different services. This distribution of services allows the implementation of contestability in each service due to the rather loose dependencies.

## 3 Results

Based on our semantic modeling concept and a prototype system, we develop a proof of concept. To do so, we first adapt general contestability requirements to the concept in Section 3.1. Then, we fine-tune language models for the three language modeling tasks, evaluate them, and present our results in Section 3.2. Finally, we present our proof of concept in Section 3.3.

### 3.1 Adjusting contestability requirements

There is a wide range of general requirements for contestability in AI systems, as presented in Section 2.1.1. We evaluate these requirements in the context of the proposed system for practicality and validity in this context and with respect to the stakeholders involved.

The first requirement, *built-in safeguards against harmful behavior*, is only partially applicable because our system has no procedural decision-making capabilities, and all decisions are provided to a human for oversight and analysis. Storing predictions and user modifications for further monitoring is a requirement that our system should implement.

*Interactive control over automated decisions* is a highly relevant requirement for human controllers in our system. In the proposed system, all model decisions must be correctable by users. This is especially important because the extraction of relationships depends on the annotation of the previous token classification step. Correcting classified tokens should therefore lead to a new predicted version of the relationship annotations, making it necessary to review and correct these relationships afterwards.

The next required feature, *explanations of system behavior*, can be provided from different perspectives. Ploug and Holm (2020) note that explaining decisions is open to interpretation. Thus, explanations of decisions range from simply stating that the AI made a decision based on some data, to explaining why a decision was the scientifically best decision based on the available features and parameters. The more precise explanations are local explanations, that approximate the influence of inputs on the outputs of a single prediction. However, for the proposed system, we decide not to use local explanations due to their complexity and limited applicability to our tasks. First, selecting an appropriate explainable AI algorithm requires careful consideration of the model, problem, and hardware constraints (Cugny et al., 2022; Krakowczyk et al., 2023). In addition, local explanations introduce significant computational overhead (Doumard et al., 2023; Bassan et al., 2024), making them impractical given that our tasks require thousands of predictions per example. Furthermore, we argue that their usefulness varies across our classification tasks. While the identification of important tokens can be useful for tracing reasons for multi-label classification of crime types, attribution scores are less informative for token classification and relationship extraction. Given the trade-off between the value of such explanations and the added complexity they introduce, we instead prioritize result visualization and confidence scores, which we consider sufficient for understanding our system and its behaviors. Another approach that falls under the category of explaining system behavior is to consider the traceability of decisions. In this case, tracing a decision could be done by logging what was predicted and what changes a user made to an example. Logging with version control to monitor examples is an approach suggested by Aler Tubella et al. (2020). For the police report analysis tool, users are encouraged to correct model predictions, resulting in multiple versions that are logged.

*Human review and intervention requests* focus on the ability of decision subjects and third parties to receive information and challenge decisions made by a system. Our system focuses on structuring unstructured information in text and does not make direct decisions about decision subjects. Therefore, the need for procedural intervention, such as that proposed in the challenge and justification protocol (Henin and Le Métayer, 2021), is questionable. Our proposed system generates thousands of classifications or predictions for each individual example. We argue that if the system allowed each decision to be challenged with justification and evidence, efficiency would be reduced and usability would be compromised. Therefore, we allow users to challenge and correct decisions, but do not require evidence for all changes. Correcting decisions can thus be done for all available forecasts.

The final contestability feature concerns *tools for scrutiny by subjects or third parties*. This includes information about system performance indicators related to training data and models, information about the system itself and the development process, and information about the decision process and the role of human decision-makers. We agree with this requirement for our system and require that information about training data, annotations, test procedures, performance evaluation results, and the embedding of the system in the decision process is provided. We do this through documentation of data, annotations, processes throughout this work, and the presentation of our performance evaluation in Section 3.2. Because this work provides a proof of concept without a real-world deployment scenario, it goes beyond the scope of implementing complete solutions for all requirements. Instead, we develop some proof of concept and suggest ideas for further development. For example, since our application stores data and usage logs, an application programming interface (API) can be provided as a tool for investigating current system usage and performance. Using the generated log data, it is possible to generate statistics about predictions and corrections per task, per dataset or subset of data, and limited by time frames.

In terms of design practices, some requirements are of limited applicability to this proof of concept because it is not intended to be used in a real-world criminal investigation scenario. For example, there are no stakeholders involved beyond the authors of this study. Therefore, *ex-ante safeguards* or *third-party oversight* are not feasible in this work. However, this does not diminish their importance in future work, where they should be evaluated and implemented accordingly. *Agonistic approaches to machine learning development*, on the other hand, are applicable. To involve humans in the decision-making process, they must be in constant interaction with the system. Therefore, we require our system to tightly integrate users by providing them with interactive exploration and modification features that naturally ensure user autonomy. In addition, quality assurance during development is critical, as our system relies on the performance of the underlying language models. Therefore, we are transparent about our data, models, and evaluation. Since there is no dedicated deployment in this study, *post-deployment quality assurance* must at least be prepared by providing features such as logging and versioning of predictions and decisions. Moreover, *risk mitigation* is done in the early stages of data annotation, where annotators are trained to make accurate annotations. It should also be done through intuitiveness and explanations in the user interface in addition to training users to use the system.

### 3.2 Language modeling tasks

As discussed earlier, contestability requires, among other things, accurate information about data, models, and performance. Providing this information makes a system transparent to stakeholders. In addition, accurate modeling is critical to the functioning and quality of the system. Our proposed system relies on language models to extract information in a standardized format. We have specified three language modeling tasks: crime type classification, token classification, and relationship extraction. For these reasons, in the following we present solutions to these tasks, which involve fine-tuning and evaluation of language models. Since all three tasks are classification tasks, we use established metrics, namely precision, recall, and F1 score (Powers, 2020). They are defined as


(1) $$\begin{array}{l} \text{Precision}=\frac{TP}{TP+FP} \end{array}$$

(2) $$\begin{array}{l} \text{Recall}=\frac{TP}{TP+FN} \end{array}$$

(3) $$\begin{array}{l} F1=2\times \frac{\text{Precision}\times \text{Recall}}{\text{Precision}+\text{Recall}} \end{array}$$

In these equations, *TP* (true positives) refer to correctly predicted positive samples, *TN* (true negatives) refer to correctly predicted negative samples, *FP* (false positives) refer to incorrectly predicted positive samples, and *FN* (false negatives) refer to incorrectly predicted negative samples.

Based on the raw police report dataset, presented in Section 2.3.1, we have created three sub-datasets by annotating labels for sequence classification (see Section 2.3.2), token classification (see Section 2.3.3), and relationship extraction (see Section 2.3.4). In this Section, we use these three sub-datasets, carry out fine-tuning experiments, evaluate the performance of the language models on the validation splits of the three sub-datasets, and present the results of the best-performing models. Because of the proof of concept nature of this work, and the small dataset size we do not extensively carry out hyperparameter and model performance optimization and instead rely on standard parameters proposed by Devlin et al. (2019) and Yamada et al. (2020).

As previously shown, the sub-datasets for all three tasks exhibit label imbalance. To evaluate overall classifier performance, we report the macro- and micro-averaged F1 score, precision, and recall. To enhance interpretability, we provide 95% confidence intervals (CI) for these aggregated metrics using bootstrap resampling. In addition, we report label-wise F1 score, precision, and recall to highlight performance on individual classes. CIs for label-wise metrics are omitted to improve readability.

#### 3.2.1 Crime type classification

Crime type classification, introduced in Section 2.3.2, is a multi-label sequence classification task. We selected several pretrained encoder models for fine-tuning to this classification task because they are established in existing research with strong performance in various classification tasks. The best performing model is a German BERT model, *deepset/gbert-large* proposed by Chan et al. (2020), which is pre-trained on German data and gives strong results on several existing benchmarks. We used the raw police report without any additional preprocessing. The results are presented in Table 5 and show a strong performance for all labels except the *Other* class. While the different results may be due to the distribution of the labels, the *Other* category includes reports with different and less clearly defined topics, making it more difficult for the model to learn a consistent representation of this class.

**Table 5:** Evaluation results of the fine-tuned multilabel crime type classification model (*deepset/gbert-large*) on the validation dataset.

**Label**	**F1-Score (CI)**	**Precision (CI)**	**Recall (CI)**
Macro	0.83	0.89	0.78
	(0.79–0.86)	(0.85–0.93)	(0.74–0.82)
Micro	0.84	0.91	0.78
	(0.82–0.87)	(0.88–0.94)	(0.74–0.82)
Drugs	0.99	0.99	0.98
Weapons	0.86	0.86	0.86
Other	0.66	0.82	0.55
Danger to life and limb	0.80	0.89	0.73

Macro and micro averages are reported with 95% CIs obtained via bootstrap resampling, shown in the format: mean (lower–upper). Per-label results are shown without bootstrap CIs for improved readability.

#### 3.2.2 Token classification

The token classification task, introduced in Section 2.3.3, requires a classification for each token in the input sequence. Since the *PERSON* class is irrelevant for this particular dataset, and the *TIME* class is predicted by the rule-based library, we trained models using the *COUNT, LOCATION, OBJECT, ORGANIZATION, PERSON-REFERENCE*, and *PROPERTY* classes. To combine multiple tokens into a span, we used the IOB scheme invented by Ramshaw and Marcus (1995). This tagging scheme suggests three general token types. The O-tag, for outside, marks all tokens that do not belong to a span. The B-tag, for beginning, marks the first token of a span. For spans containing multiple tokens, the I-tag, for inside, marks all tokens within a span after the first token.

Using the labeled and preprocessed data, we took 90 % of the data for training and 10 % for validation. After comparing several language models, a pre-trained multilingual LUKE model, *studio-ousia/mluke-large* proposed by Yamada et al. (2020), performed best on this dataset. The results of the validation split are presented in Table 6 and show a strong performance across all labels, with the *PROPERTY* class performing the worst.

**Table 6:** Evaluation results of the fine-tuned token classification model (*studio-ousia/mluke-large*) on the validation dataset.

**Label**	**Tag-Type**	**F1 Score (CI)**	**Precision (CI)**	**Recall (CI)**
Macro	-	0.86	0.87	0.85
		(0.85–0.87)	(0.86–0.88)	(0.84–0.86)
Micro	-	0.96	0.96	0.96
		(0.96–0.96)	(0.96–0.96)	(0.96–0.96)
O-Tag	O	0.98	0.98	0.98
LOCATION	B	0.76	0.76	0.76
	I	0.83	0.91	0.77
ORGANIZATION	B	0.93	0.92	0.94
	I	0.86	0.90	0.82
PERSON-REFERENCE	B	0.92	0.93	0.91
	I	0.92	0.95	0.89
OBJECT	B	0.86	0.86	0.87
	I	0.87	0.89	0.86
COUNT	B	0.86	0.88	0.84
	I	0.89	0.93	0.84
PROPERTY	B	0.70	0.63	0.79
	I	0.76	0.71	0.81

Macro and micro averages are reported with 95% CIs obtained via bootstrap resampling, shown in the format: mean (lower–uper). Per-label results are shown without bootstrap CIs for improved readability.

#### 3.2.3 Relationship extraction

The final modeling task is relationship extraction, introduced in Section 2.3.4. Again, we use a multilingual LUKE model for fine tuning. A LUKE model is given the input text and the positions of two spans for which a relationship is to be classified. The model does not receive any information about the span classes, which requires the model to rely solely on the learned semantic representation of such words (Yamada et al., 2020). Furthermore, due to the behavior of receiving two spans at a time, the model can only classify one relationship per input example. Let *s* be the number of spans extracted from the previous step, token classification, and *R*(*s*) be the number of possible relationships between these spans. This leads to the requirement of predicting each text *R*(*s*) times with different span combinations for each of the possible relationships within a text. This fact increases the computational complexity of classifying all relationships within a report as the number of spans increases. Given a text containing spans, each possible relationship is formed between two different spans. As a result, the number of possible relationships is given by the binomial coefficient:


(4) $$R(s)=\left(\begin{array}{c} s\\ 2 \end{array}\right)=\frac{s!}{2!(s-2)!}=\frac{s(s-1)}{2}.$$

For large values of *s*, the significant part of this expression is proportional to *s*^2^, leading to quadratic complexity:


(5) $$\begin{array}{l} R\left(s\right)=O\left(s^{2}\right). \end{array}$$

This means that the number of relationship classifications grows quadratically with the number of spans in a given text. This complexity can lead to high computational requirements, especially for long texts with a higher number of extracted spans. To reduce the computation time, we filter invalid span combinations before classifying relationships. As mentioned above, there are certain span combinations that allow a relationship to exist. The valid head and tail span combinations are listed in Table 3.

Using this algorithm for preprocessing the examples, we trained the *studio-ousia/mluke-large* model on the relationship extraction task. The results on the validation set are presented in Table 7. While the macro average F1 score is 0.77, the metric results demonstrate significant variation across the possible relationship labels. The performance for the *COUNTS* relationship exhibits the best results. This may be due to the fact that this is the only relationship that originates from the *COUNT* span. Although *PROPERTY* spans are the only spans from which the *BELONGSTO* relationship originates, and there are almost twice as many *BELONGSTO* relationships as *COUNTS*, the F1 score is 0.2 points lower. The model performs worst on the *CONTAINS* and *STAY* relations. Here, both the complexity of the semantic relations and the limited amount of available training data could be reasons for this performance.

**Table 7:** Evaluation results of the fine-tuned relationship extraction model (*studio-ousia/mluke-large*) on the validation dataset.

**Label**	**F1 Score (CI)**	**Precision (CI)**	**Recall (CI)**
Macro	0.77	0.77	0.77
	(0.76–0.78)	(0.76–0.78)	(0.76–0.78)
Micro	0.96	0.96	0.96
	(0.96–0.96)	(0.96–0.96)	(0.96–0.96)
BELONGSTO	0.74	0.74	0.75
CONTAINS	0.52	0.55	0.49
COREF	0.85	0.81	0.89
COUNTS	0.97	0.97	0.97
NONE	0.98	0.98	0.98
STAY	0.55	0.56	0.54

Macro and micro averages are reported with 95% CIs obtained via bootstrap resampling, shown in the format: mean (lower–upper). Per-label results are shown without bootstrap CIs for improved readability.

### 3.3 Proof of concept

Previously, we have designed a pipeline for semantic modeling of police reports, conceptualized a prototype system for users to interact with the data and pipeline results in Section 2.3, adapted contestability requirements to the concept of this system in Section 3.1, and evaluated the information extraction results in Section 3.2. The final step is to implement a proof of concept that combines the information extraction in a prototypical system while satisfying the contestability requirements. To do so, we first describe the technical architecture of our implementation, then explain the user interface, and finally outline the implemented contestability solutions.

As mentioned before, we decided to implement a distributed system consisting of several interacting services. The first service is a *relational database* that serves as the foundation and stores the semantic models, including the police report texts, metadata, annotations, and specific entity information. The second service, an *application backend*, handles the business logic, database interaction, and API endpoints. This service handles most of the processing involved in the semantic modeling pipeline shown in Figure 1. For example, it handles queries to geolocation services for identified *LOCATION* spans, or creates a graph representation of all the relationships in an example and merges coreferences. Since language model deployment is most performant with GPU acceleration, the third service is a *model inference service* with GPU access, that provides API endpoints to our best models for the three language modeling tasks. This API is used by the *application backend* whenever one of the three tasks must be predicted. The fourth and final service provides the user interface in a web browser via a *frontend web application*.

**Figure 4:**
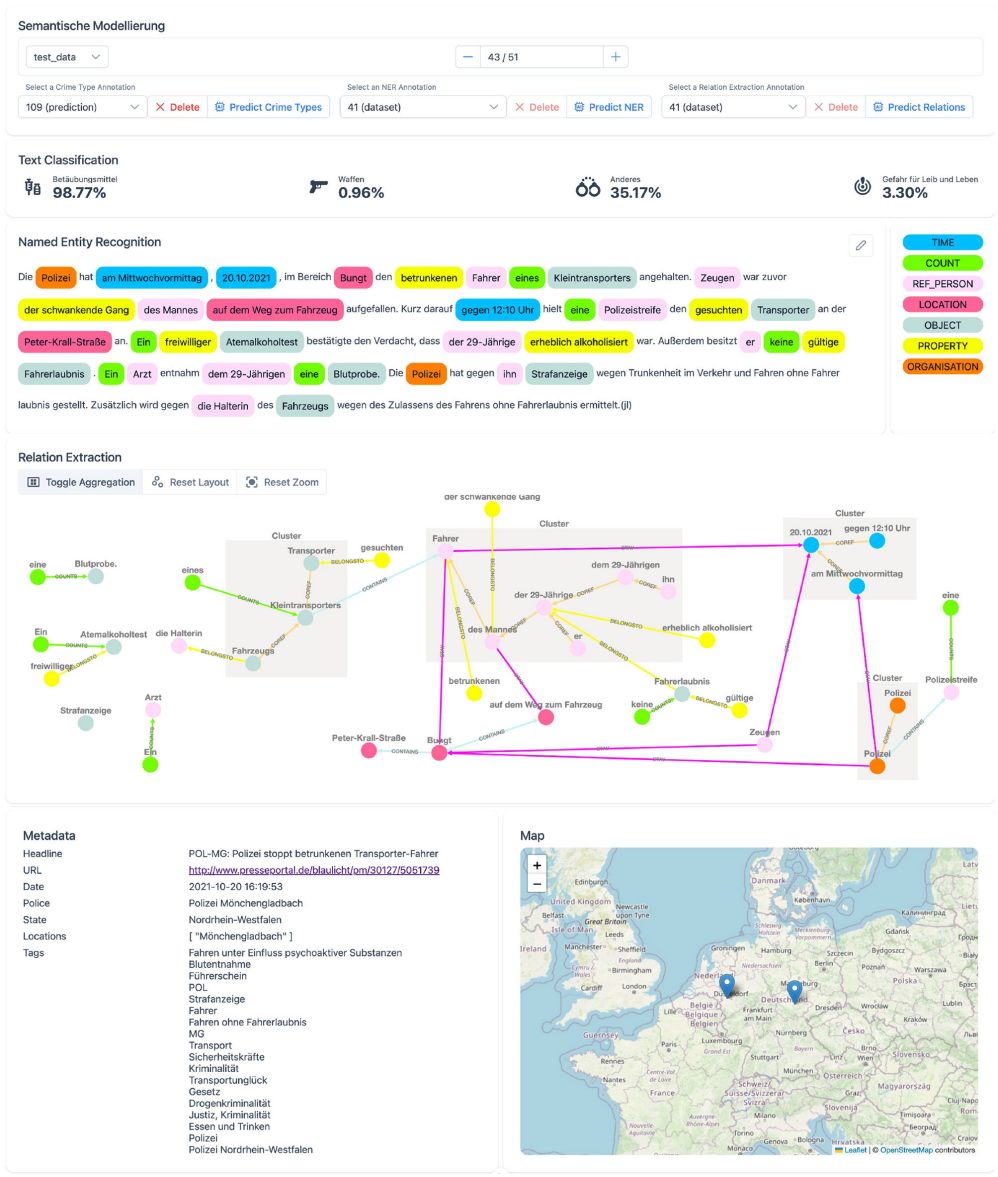
User interface of the implemented proof of concept system.

The user interface, as shown in Figure 4, is a reactive web page that allows users to interact with semantic models. Information is presented in simple, bordered components that separate tasks from one another and help the user to focus on one task at a time. The first component of the user interface provides data selection functionality. The user can select an example from different datasets, select or remove an existing version of a task annotation, or predict a new version. The second component visualizes the classification of crime types. Manual annotations are displayed as Boolean values, and predicted annotations also display sigmoid scores, which can be interpreted as confidence scores for each class. The component for token classification shows the classified spans in the text. The accompanying legend explains the colors and their corresponding classes. Selecting classes in the legend also highlights the selected classes in the text, allowing the user to easily navigate through the report. Classified spans can be clicked to open a detailed information overlay. This overlay component displays all tokens within an entity, all inbound and outbound relationships, and other detailed information. Detailed information includes extracted time information for *TIME* spans or geolocation information with a plotted map for *LOCATION* spans. Relationships between spans are visualized in the graph component, which displays all spans as nodes and uses class-labeled arrows to indicate relationships between nodes. To visualize coreference clusters, the aggregation mode merges all related coreference nodes into a single cluster and connects relationships of included nodes to the cluster. In addition, nodes or spans hovered in either component highlight the node in both components. The last two components display the metadata and a map. The map shows all extracted geolocations.

Contestability features are built into several components of the system. The first requirement of challenging AI decisions is implemented for all language modeling tasks. For crime type classification, users can override predictions to change the Boolean value of a crime type label. For token classification, the component can be used in an edit mode. This allows users to remove predictions or add missing spans, which are then persisted. Additionally, relationships can be edited in the graph component to remove an existing relationship or add a new relationship between two spans. Here, two nodes are selected as source and target nodes before a relationship can be selected, which ensures that only valid relationships are created, as shown in Table 3. Since the predictions are suggestions to the user, they should be verified and corrected through iterative editing. This process involves verifying and correcting the token classifications before requesting, verifying, and correcting new predictions for relationship annotations.

## 4 Discussion

This work has combined the development of an AI-based criminal intelligence analysis system with contestability requirements. For this purpose, an information extraction pipeline for semantic modeling of police reports was conceptualized, and the system was designed by adapting contestability requirements and implementing appropriate solutions.

We have raised **RQ2** about how police reports can be modeled to support investigative work. To answer this question, we have proposed a semantic modeling pipeline that includes three language modeling tasks, namely crime type classification, token classification, and relationship extraction. Combining these steps with metadata, external knowledge, and sophisticated postprocessing provides structured information in a unified data format. This semantic model provides structured insight into the storyline, actors, locations, and times. In addition, this data can be used to explore individual examples with intuitive visualizations or to automatically analyze large datasets. Automatic analysis uses the structured data to filter for specific crime types, specific locations, specific time periods, or can be used to generate management reports. Furthermore, data analysis can provide useful information for cross-referencing with other investigative results. Here, the combination of multiple investigative results, databases, and our semantic model could lead to finding missing pieces of a puzzle to solve an investigative case. The proof-of-concept system we have developed relies on the performance of the underlying language models, which are trained in a supervised manner on manually annotated data. Although our results show strong performance for most classification labels, there are a few labels, such as *PROPERTY* spans or *CONTAINS* and *STAY* relationships, that could not be classified with reasonable accuracy. There are three possible reasons for this: data availability, task complexity, and model capacity. Since most of the classes were modeled reasonably well, we conclude that the model capacity is sufficient. More important is the low availability of accurately annotated data. In particular, annotating labels for token classification and relationship extraction is very time-consuming and error-prone. For humans, this task first requires a learning process of available labels and how to annotate them correctly, and also requires constant concentration to read and classify each word, without accidentally skipping relevant labels. Additionally, the complexity of the labeling schemes is relevant for the accuracy of the annotations and the performance of the trained language models. For the classification of crime types, we have defined a multiclass scheme with four labels. One of them is an *Other* class, which is difficult for the model to predict accurately. With a sharper distinction of the classes, e.g., by adding more classes, a model could learn a better internal representation of the available crime types, which would lead to a better performance. The same conclusion applies to token classification and relationship extraction. As mentioned earlier, there is an almost infinite number of possible labels. For example, in future work, subcategories of objects or properties and other relationships can be added to allow for more precise descriptions of people or their behaviors.

The complexity of the labeling schemes influences the quality of semantic modeling, while the computational complexity of the approach influences the time and resources required, which greatly affects practicability. Let *n* be the number of police report examples, and for each example, let *t* be its token count. For each example, our pipeline sequentially executes three language modeling tasks. The first task, sequence classification, processes the entire input of *t* tokens and produces a single prediction. While treating the model's encoding procedure as a black box, its per-example complexity is constant, *O*(1). For the full dataset, this complexity is *O*(*n*). The second task, token classification, assigns labels to each token, yielding a complexity of *O*(*t*) per example and *O*(*nt*) overall. The third task, relationship extraction, operates on all pairs of extracted spans from the token classification step. In the worst case, the number of spans *s* is proportional to the number of tokens (*s* = *t*). Since the relation extraction model predicts each possible combination of spans, this leads to a complexity of *O*(*s*^2^) = *O*(*t*^2^) per example and *O*(*nt*^2^) over the dataset. Consequently, the overall worst-case complexity for the pipeline is *O*(*nt*^2^), which leads to the requirement of costly hardware acceleration. Although using powerful hardware can improve practicability and processing speed, a more complex labeling scheme would lead to a more complex annotation process, a more appropriate semantic model for police reports, an arguably more complex language modeling task, and further analysis capabilities. Therefore, the goal is to find the optimal balance between an accurate but complex semantic model and a simple task for reasonable performance. Future research should not only explore approaches to handle more complex labeling schemes, but also more efficient language models. Possible approaches include, but are not limited to, using generative language models to generate labels before fine-tuning smaller models, or fine-tuning more complex models. In addition, future work could explore how this system can be connected to other policing systems, open source intelligence data, or internal databases, which could improve the accuracy of information extraction and lead to deeper analysis capabilities.

The main research question we have introduced is **RQ1**: How do contestability requirements have to be adapted to fit an AI-based criminal intelligence analysis system? Based on the semantic modeling pipeline, we have designed a prototype system for analyzing police reports. To make this system contestable, we first presented an overview of existing general contestability requirements for systems and system design, before considering these requirements for the development of our prototype system. Based on the intended use and stakeholders of our system, and using the contestability terminology of Alfrink et al. (2023), we discussed the applicability of the requirements in this context. With these refined contestability requirements, we implemented a proof-of-concept system that provides contestability for criminal intelligence analysis of police reports. This adaptation for our use case of criminal intelligence analysis focused on *information provision, interactive controls*, and *quality assurance*. The implemented system thus integrates intuitive visualizations through the use of reactive components, a consistent color scheme, and graph views. These provide users with all relevant information to exercise their right to contest. Furthermore, the system provides several features to correct and modify annotations, which are partially predicted by language models. Regarding the requirement for *explanations of system behavior*, we decided not to focus on local explanations for individual decisions because of the large number of individual predictions made for each example. Furthermore, we argue that local explanations for our tasks do not provide precise reasoning for the implemented tasks, and that they overwhelm users. Instead, we focus on intuitive visualizations that display confidence scores and provide information for human controllers to make responsible decisions. Although we did not choose to require explanations for individual decisions, this requirement can be evaluated in future research. In addition, we leave for future research a qualitative evaluation of the contestability requirements and related solutions implemented in our system. Studies could look at how users work with the system, how such a system can be connected or adapted to other criminal investigation data, or how decision subjects are influenced by such a system and how they can be provided with contestability.

Semantic modeling is generally adaptable to a wide range of text genres. However, our labeling schemas are designed specifically for German police reports, which exhibit characteristics such as the exclusion of personal names. These schemas should not be adopted uncritically for other corpora. New text types would likely require revised schemas, as well as the collection and annotation of new training data. Although our pipeline, database, and model architectures can be retained and reconfigured to accommodate these new schemas, it may be advantageous to start from different pre-trained model checkpoints, particularly ones better suited to the target language or domain. At the same time, the contestability requirements we imposed inherently constrain transferability. For instance, in the context of medical report analysis, our semantic modeling pipeline might fail to extract the fine-grained details needed, and our proposed user interface might lack the specialized visualization and correction tools required by clinicians. Nevertheless, by laying the groundwork for contestability adaptation in this work, we have established solutions that can largely be reused. However, depending on the context, stakeholder groups, data, and analytical objectives, additional modifications and complementary features will be necessary to meet new requirements.

In conclusion, in this work we have proposed a contestable AI system for criminal intelligence analysis of police reports that uses a novel information extraction pipeline for semantic modeling. Based on three language modeling tasks with custom labeling schemes, we created a dataset, trained language models with promising results, and implemented a system for users to interact with the data and challenge all decisions within a pipeline. This work thus expands the scope of information extraction in police report analysis by expanding the range of data types that can be automatically identified and analyzed, ultimately leading to a more transparent and reliable AI-driven investigation with greater depth.

## Data Availability

The datasets presented in this article are not readily available because the dataset used in this work is proprietary and was used for a proof of concept only. Therefore, the data cannot be published. Requests to access the datasets should be directed to Falk Maoro, falk.maoro@unibw.de.
